# Interleukin-6: The Unexpected Opponent to Pancreatic Cancer

**DOI:** 10.1016/j.jcmgh.2026.101799

**Published:** 2026-04-30

**Authors:** Gregory B. Lesinski

**Affiliations:** Department of Hematology and Medical Oncology, Winship Cancer Institute of Emory University, Atlanta, Georgia

Cytokines are instrumental in shaping the biology of tumors and directly impact whether the immune system supports or eliminates the malignancy. Given their prominent influence, several efforts are ongoing to explore the therapeutic targeting of these soluble immune mediators. Although cytokine dysregulation is a feature of aggressive tumors, clearly delineating their role can be a challenge for several reasons. First, these potent factors are derived from multiple cell populations in the tumor microenvironment, making their origins heterogeneous. Second, the signaling events induced downstream of cytokines is, by design, redundant, making it a challenge to delineate the most physiologically dominant mediators. Finally, their potent action induces negative regulatory cascades, to balance inflammatory responses, and is geared at achieving homeostasis. Together, these considerations create a challenge for definitive understanding as to the role of cytokines in cancer and underscore the need for careful study.

One cytokine of particular importance in modulating tumor-immune interactions is interleukin-6 (IL-6). This cytokine has been studied comprehensively in the setting of pancreatic ductal adenocarcinoma (PDAC), an aggressive malignancy that is typically refractory to both chemotherapeutic and immunotherapy regimens.^1^ It is well-established that IL-6 is elevated in PDAC tumors and can act on both malignant cells to promote their progression to metastasis.^2^ This cytokine can be derived from tumor cells, cancer-associated fibroblasts, and other immune cells present in these tumors. It is also a powerful immunomodulatory factor, driving T cells to distinct phenotypes, and altering the function and survival of antigen-presenting cells.^3^^,^^4^ These results have catalyzed several efforts to target IL-6 and its associated signaling pathways. In preclinical studies, IL-6 pathway blockade improves the efficacy of immune checkpoint blockade in a manner dependent on T cells.^5^^,^^6^ These combinations are currently being evaluated in patients with PDAC, among other tumor types, in early phase clinical trials. A recent randomized clinical trial in patients with PDAC further indicates that IL-6 receptor blockade can be safely combined with chemotherapy. Although the trial did not meet its efficacy endpoint, there was evidence that this treatment may be associated with reduced muscle wasting.^7^

In this report by Arneson-Wissink et al, the authors demonstrate how engineering pancreatic cancer cell lines to over-express IL-6 elicits T cell–mediated control of tumors in immune-competent murine models.^8^ This work offers provocative and novel data that establish super physiologic levels of IL-6 in PDAC can engage immune recognition and, ultimately, elimination of these tumors. The findings of this study are innovative and challenge the existing paradigm that IL-6 is exclusively a supportive cytokine in aggressive pancreatic tumors. One strength of the study in particular is the use of immune-competent orthotopic models, which better approximate the organ site in human PDAC tumors. Certainly, the results from this study offer a more nuanced, context-dependent role for IL-6 that invites further study and adaptation for treatment.

Although initially surprising, these data are aligned with other published work documenting how IL-6 can enhance distinct aspects of antitumor immune responses. For example, T cell–specific deletion of the IL-6 receptor alpha chain results in impaired Th1 and Th17 T cell responses in vivo. These data indicate that IL-6 signaling is key for CD4+ T cell memory formation.^9^ This requirement for IL-6 in T cell memory and, ultimately, antitumor activity was also evident in adoptively transferred T cells that were polarized to a Th17 phenotype.^10^

Overall, these results highlight the complexity of cytokine signaling in PDAC and leave open the opportunity to address further questions. In particular, the threshold of IL-6 that dictates the balance between tumor promotion and elimination remains a mystery. This information would be needed if translating subsequent gene therapy to patients. Further, it remains unclear whether supraphysiologic IL-6 would impact PDAC liver metastasis, whereby this cytokine and its associated signaling pathways are pivotal for the acute phase response and a prometastatic niche.^11^^,^^12^
